# Post-transcriptional Regulation of BRCA2 through Interactions with miR-19a and miR-19b

**DOI:** 10.3389/fgene.2016.00143

**Published:** 2016-08-31

**Authors:** Elena Mogilyansky, Peter Clark, Kevin Quann, Honglei Zhou, Eric Londin, Yi Jing, Isidore Rigoutsos

**Affiliations:** ^1^Computational Medicine Center, Sidney Kimmel Medical College, Thomas Jefferson University, PhiladelphiaPA, USA; ^2^Department of Pathology and Laboratory Medicine, The Children’s Hospital of Philadelphia, PhiladelphiaPA, USA; ^3^Sidney Kimmel Medical College, Thomas Jefferson University, PhiladelphiaPA, USA

**Keywords:** BRCA2, cancer, DNA repair, microRNA, the miR-17/92 cluster, miR-19a, miR-19b, post-transcriptional regulation

## Abstract

Breast cancer type 2, early onset susceptibility gene *(BRCA2)* is a major component of the homologous recombination DNA repair pathway. It acts as a tumor suppressor whose function is often lost in cancers. Patients with specific mutations in the *BRCA2* gene often display discrete clinical, histopathological, and molecular features. However, a subset of sporadic cancers has wild type *BRCA2* and display defects in the homology-directed repair pathway, which is the hallmark of ‘BRCAness.’ The mechanisms by which BRCAness arises are not well understood but post-transcriptional regulation of *BRCA2* gene expression by microRNAs (miRNAs) may contribute to this phenotype. Here, we examine the post-transcriptional effects that some members of the six-miRNA cluster known as the miR-17/92 cluster have on the abundance of *BRCA2*’s messenger RNA (mRNA) and protein. We discuss two interactions involving the miR-19a and miR-19b members of the cluster and the 3′UTR of *BRCA2*’s mRNA. We investigated these miRNA:mRNA interactions in 15 cell lines derived from pancreatic, breast, colon, and kidney tissue. We show that over-expression of these two miRNAs results in a concomitant decrease of *BRCA2’s* mRNA and protein expression in a subset of the tested cell lines. Additionally, using luciferase reporter assays we identified direct interactions between miR-19a/miR-19b and a miRNA response element (MRE) in *BRCA2*’s 3′UTR. Our results suggest that BRCA2 is subject to a complex post-transcriptional regulatory program that has specific dependencies on the genetic and phenotypic background of cell types.

## Introduction

The DNA repair pathway functions to correct potential damages to the chromosomes that occur during DNA replication or upon insult by environmental factors. Alterations of this pathway have been associated with many different diseases, most notably cancers (Gavande et al., 2016). Among the genes participating in this pathway, *BRCA2* is one of the best-studied (Cleton-Jansen et al., 1995; Goldgar et al., 1995; Wooster et al., 1995; Venkitaraman, 2002; Wooster and Weber, 2003; Turner et al., 2004; Prakash et al., 2015). BRCA2 is essential for the support of chromosomal integrity, and functions as an effector of HDR of DSDB and stalled replication forks (Moynahan et al., 2001; Lomonosov et al., 2003; Schlacher et al., 2011). BRCA2 functions during embryogenesis and normal development and is expressed in all tissues with some of the highest levels found in breast and thymus (Roy et al., 2012).

Human *BRCA2* has two known protein-coding transcripts with lengths of 11,986 and 10,984 nucleotides (nts), respectively. Both mRNAs code for a large protein (384,225 Da) that comprises 3,418 amino acids (Venkitaraman, 2002; Roy et al., 2012). BRCA2 does not show substantial sequence similarities to other proteins and has been co-evolving with BRCA1 (Lou et al., 2014). The amino acid sequence of BRCA2 shows poor conservation among vertebrates (Prakash et al., 2015) with 59.2% identity between human and mouse (Connor et al., 1997) and 37% identity between human and chicken (Warren et al., 2002). By comparison, other tumor suppressors such as MSH, XPA, and TP53 are much better conserved with the human and mouse sequences exhibiting 92, 86, and 77% similarity, respectively (Jasin, 2002).

In cancers, *BRCA2* functions as a tumor suppressor gene (Bieche and Lidereau, 1995; Collins et al., 1995). Similarly to *BRCA1*, *BRCA2* is haploinsufficient: mutation of one copy of *BRCA2* results in hereditary autosomal-dominant breast and ovarian cancer syndrome (Roy et al., 2012). Also *BRCA2* germline mutations increase one’s risk to develop pancreatic, stomach, laryngeal, fallopian tube, or other cancers. *BRCA2* germline mutations are the most frequent genetic alteration in familial pancreatic cancer and occur in 5–20% of the patients (Murphy et al., 2002; Hahn et al., 2003; Couch et al., 2007; Dagan, 2008; Ferrone et al., 2009; Ghiorzo et al., 2012; Luo et al., 2015; Zhen et al., 2015). Additionally, *BRCA2* mutations occur in 3.6–10% of all sporadic pancreatic cancers (Goggins et al., 1996; Ozcelik et al., 1997; Figer et al., 2001; Ferrone et al., 2009; Lucas et al., 2013; Luo et al., 2015). Recent therapeutic strategies using platinum salts and poly (ADP-ribose) polymerase (PARP) inhibitors exploit the presence of *BRCA1*/*BRCA2* mutations in the synthetic lethality concept (Bryant et al., 2005; Farmer et al., 2005; Helleday et al., 2005).

Tumors with *BRCA1/2* mutations exhibit specific phenotypes. Recent evidence has revealed that some sporadic forms of cancers exhibit similar molecular, histological, and clinical phenotypes even *in the absence* of *BRCA1/2* mutations, a defect known as ‘BRCAness,’ whose mechanism is not well understood (Turner et al., 2004; Lord and Ashworth, 2016). As much as 25% of sporadic breast and ovarian cancers exhibit this phenotype (Cancer Genome Atlas Research Network, 2011, Banerji et al., 2012; Cancer Genome Atlas Network, 2012; Stephens et al., 2012; Patch et al., 2015). Additionally, lung, prostate, and pancreatic cancer also exhibit BRCAness phenotype (Couch et al., 2007; Beltran et al., 2013; Holter et al., 2015; Lord and Ashworth, 2016). The phenotype renders tumors sensitive to platinum salts and PARP inhibitors (Bryant et al., 2005; Farmer et al., 2005; Helleday et al., 2005).

MiRNAs are short (∼22 nt in length) ncRNAs that act as post-transcriptional regulators and are typically derived from endogenous hairpin-like transcripts (Bartel, 2004, 2009). MiRNAs regulate the abundance of both protein-coding genes and ncRNAs by inhibiting protein translation or through mRNA degradation (He and Hannon, 2004; Filipowicz et al., 2008). Since the discovery of the first animal miRNA, lin-4, in *C. elegans* (Ambros, 1989; Ruvkun and Giusto, 1989), miRNAs have been characterized in numerous animal species and plants, and several viruses (Kozomara and Griffiths-Jones, 2014). The public repository of miRNA sequences known as miRBase (Kozomara and Griffiths-Jones, 2014) lists 2,588 human mature miRNAs in its latest release. However, in a recent report, by analyzing data from only 13 human tissues, we reported an additional 3,707 human miRNAs, which suggests that the eventual number of human miRNAs is likely to be higher (Londin et al., 2015).

MiRNAs are involved in the regulation of many different biological processes, including proliferation, apoptosis, DNA repair, and many others. Their aberrant expression has been associated many diseases and disorders including cancer (Calin et al., 2004; Meng et al., 2007; Peng et al., 2011; Fang et al., 2012; Huang et al., 2014; Misso et al., 2014), cardiovascular (Corsten et al., 2010; Fichtlscherer et al., 2010; Zampetaki and Mayr, 2012; Philippen et al., 2015), immune-related (Stanczyk et al., 2008; Junker et al., 2009; Lofgren et al., 2012; Nielsen et al., 2012; Liu et al., 2014; Yang et al., 2014), neurodegenerative conditions (Nunez-Iglesias et al., 2010; Margis et al., 2011; Martins et al., 2011; Femminella et al., 2015) and other diseases (Starczynowski et al., 2010; Wu et al., 2010; Brest et al., 2011). MiRNAs are also involved in the DNA repair pathway: for example, miR-24, miR-421, and miR-21 modulate the expression of multiple DDR genes such as *H2AX*, *ATM*, and *CDC25A*, respectively (Hu and Gatti, 2011); miR-1245, miR-1255b, and miR-193b^∗^ were shown to target BRCA2 (Song et al., 2012; Choi et al., 2014); and, we recently reported that the miR-15/107 group of miRNAs targets BRCA1 (Quann et al., 2015).

A miRNA exerts its function by targeting sites within transcripts that are known as ‘miRNA response elements’ or MREs. The interaction of a miRNA with an MRE occurs in a sequence-depended manner (Lee et al., 1993; Reinhart et al., 2000; Bartel, 2004; Fabian et al., 2010; Xia et al., 2012). According to the canonical model, (1) MREs are located within the 3′UTR of the targeted mRNA transcript (Wightman et al., 1993); (2) within the ‘seed’ region, which spans positions 2–7 inclusive from the miRNA’s 5′ end, bases form Watson–Crick pairs (Reinhart et al., 2000; Bartel, 2004); and, (3) miRNA targets tend to be conserved across species (Friedman et al., 2009). However, previous work by others and us has been generating evidence in support of an expanded model of miRNA targeting where one or more of these three ‘rules’ are not satisfied (Wightman et al., 1993; Ha et al., 1996; Easow et al., 2007; Baek et al., 2008; Selbach et al., 2008; Tay et al., 2008; Lal et al., 2009; Hafner et al., 2010; Thomas et al., 2010; Zisoulis et al., 2010; Chi et al., 2012; Skalsky et al., 2012; Zhou and Rigoutsos, 2014; Quann et al., 2015).

The miR-17/92 cluster, also known as ‘oncomiR-1’ (He et al., 2005), comprises six miRNAs (miR-17, miR-18a, miR-19a, miR-19b-1, miR-20a, and miR-92) and is an important regulator in health (Marcelis et al., 2008; Ventura et al., 2008; Carraro et al., 2009; Grillari et al., 2010; Jiang et al., 2011) and disease (Tsitsiou and Lindsay, 2009; Yu et al., 2010; Morimura et al., 2011; Tsuchida et al., 2011; Angerstein et al., 2012; Diehl et al., 2012; Heegaard et al., 2012; Schonrock et al., 2012; Diaz-Beya et al., 2013). For recent reviews, see (Mogilyansky and Rigoutsos, 2013; Fuziwara and Kimura, 2015; Khuu et al., 2016).

In this work, we combined computational miRNA target predictions of candidate miRNAs that potentially interact with *BRCA2’s* mRNA with experimental work. Specifically, we focused our analysis on the role of two miRNAs, miR-19a and miR-19b, the members of the miR-17/92 cluster, and their interactions with *BRCA2* for the following reasons. First, these two members of the miR-17/92 cluster are known to be sufficient for recapitulating the oncogenicity of the whole cluster (Mu et al., 2009; Olive et al., 2009; van Haaften and Agami, 2010). Second, miR-19a and miR-19b are over-expressed in several cancers, including pancreatic and breast cancers (Mogilyansky and Rigoutsos, 2013). Third, in the primary screening of the miR-17/92 cluster members and putative five MREs, miR-19a and miR-19b show the greatest statistically significant decrease of the luciferase activity. Fourth, RNA-seq datasets (Londin et al., 2015) show that miR-19a and miR-19b increase from normal pancreas to early stage pancreatic cancer and then increase again from early stage to late stage of the disease. Fifth, RNA-seq datasets (Londin et al., 2015) show that miR-19a and miR-19b are increased in pancreatic cancer cell lines compared to the normal epithelial pancreatic cell line hTERT-HPNE. All of the above provide the rationale to focus on the miR-19a and miR-19b for this study. We examined the effects of these two miRNAs on *BRCA2* mRNA and protein expression and present our findings in a variety of cancerous and non-cancerous model cell lines from pancreatic, breast, kidney and colon tissues.

## Materials and Methods

### Identification of miRNA Targets

MiRNA targets were computationally predicted using RNA22 (Miranda et al., 2006). Candidate MREs were permitted along the entire length of *BRCA2’s* mRNA, i.e., in the 5′UTR, the amino acid coding sequence (CDS), and in the 3′UTR. Additionally, putative miRNA:mRNA interactions were allowed to include *non*-Watson–Crick base pairings and/or bulges in the seed region of the corresponding heteroduplexes. The candidate interactions were filtered further and only those with experimental support from our mining of public and in-house datasets generated through crosslinking followed by Argonaute (Ago) immunoprecipitation and deep sequencing (Ago CLIP-seq; Yano et al., 2010; Kishore et al., 2011; Leung et al., 2011; Loeb et al., 2012) were kept (Clark et al., 2014).

### Cell Lines

Human cell lines hTERT-HPNE, PL45, MIA PaCa-2, BxPC-3, PANC-1, AsPC-1, Capan-2, MDA-MB-231, MDA-MB-468, BT-20, MCF7, MCF-10A, and HCT 116 were obtained from the American Type Culture Collection (ATCC; Manassas, VA, USA). 239T cells were obtained from Thermo Fisher Scientific (Pittsburg, PA, USA). The PL-5 cells were a kind gift from Dr. Jonathan Brody (Thomas Jefferson University, Philadelphia, PA, USA). All cell lines were cultured using standard techniques and conditions recommended by the manufacturer’s protocols.

### Reagents

MiRNA precursors to hsa-miR-19a-3p (miR-19a), hsa-miR-19b-3p (miR-19b), and anti-miRs, anti-miR-19a-3p (anti-miR-19a), anti-miR-19b-3p (anti-miR-19b), negative controls for miR- and anti-miR precursors, and positive control BRCA2-specific siRNA (siBRCA2) were obtained from Ambion/Thermo Fisher Scientific.

### Quantitation of Expression

Cells were seeded in 6-well plates with 50% confluency and transfected 24 h later with 50 nM of miRNA- or anti-miRNA precursors according to manufacturer’s instructions. Cells were also transfected with the relevant negative and positive controls in parallel. At 48 h post-transfection, total RNA was extracted from cells using TRIzol (Thermo Fisher Scientific). cDNA synthesis was performed with SuperScript III Reverse Transcriptase (Invitrogen/Thermo Fisher Scientific) with presence of Random Primers (Invitrogen/Thermo Fisher Scientific). *BRCA2* qRT-PCR was performed with SYBR Select Master Mix (Thermo Fisher Scientific) and 25 ng of cDNA templates. Primer sequences used for qRT-PCR are shown in **Supplementary Table S1**. The human *GAPDH* RNA was amplified in parallel as an internal control. Samples were amplified on a StepOnePlus Real-Time PCR System (Applied Biosystems/Thermo Fisher Scientific). Relative gene expression levels were calculated by the ΔΔCT method with normalization to *GAPDH*. All data for qRT-PCR assays are expressed as mean ± SE with a sample size of *n* = 3 for each group. Statistical analyses between test and control group were performed with unpaired two-tailed Student’s *t*-test assuming equal variances. *P*-values ≤ 0.05 were considered statistically significant. ^∗^*P* ≤ 0.05, ^∗∗^*P* ≤ 0.01, ^∗∗∗^*P* ≤ 0.001.

For WB, cells were harvested 72 h post-transfection and re-suspended in Pierce RIPA lysis buffer (Thermo Fisher Scientific) containing complete protease inhibitors cocktail (Roche, Basel, Switzerland). Cells were then incubated on ice for 30 min with brief vortexing. The cellular debris was pelletized by centrifugation at 10,000 × *g* for 10 min and protein concentrations determined using Pierce BCA Protein Assay Kit (Thermo Fisher Scientific). Equal amount of lysates (10 μg) were separated on 6–12% sodium dodecyl sulfate-polyacrylamide gel (SDS-PAGE) at 120 V. Electrophoretic transfer to nitrocellulose membranes (GE Healthcare, Little Chalfont, Buckinghamshire, UK) was performed overnight at 4°C at 25 V. All membranes were blocked in 5% non-fat dry milk (Thermo Fisher Scientific) before overnight incubation with primary antibody in 1% Tris-buffered saline Tween-20 (TBST; Thermo Fisher Scientific) with 5% bovine serum albumin (Sigma–Aldrich, St. Louis, MO, USA) at the manufacturer’s recommended dilution at -2 to -8°C. The membranes were washed and incubated with appropriate secondary antibody in 1% TBST (Thermo Fisher Scientific) with 5% non-fat dry milk for 1 h at room temperature. The signal was developed using SuperSignal West Pico Chemiluminescent Substrate (Thermo Fisher Scientific) and detected using ImageQuant LAS4000 Imaging system (GE Healthcare). The primary antibodies include: anti-BRCA2 1:2,000 dilution (ab123491; Abcam, Cambridge, MA, USA), β-actin antibody 1:1,000 dilution (3700S; Cell Signaling Technologies, Denver, MA, USA). The secondary antibodies include: HRP-linked anti-mouse IgG 1:2,000 dilution (7076, Cell Signaling Technologies) and HRP-linked anti-rabbit IgG antibody 1:2,000 dilutions (7074; Cell Signaling Technologies). Band intensities were measured using ImageJ with normalization to actin-beta (ACTB) and appropriate controls Scramble miR/Scramble anti-miR. All data for WB are expressed as mean ± SE with a sample size of *n* = 3 for each group. Statistical analyses between test and control group were performed with unpaired two-tailed Student’s *t*-test assuming equal variances. *P*-values ≤ 0.05 were considered statistically significant. ^∗^*P* ≤ 0.05, ^∗∗^*P* ≤ 0.01, ^∗∗∗^*P* ≤ 0.001.

### Luciferase Assays

Cells were seeded in 96-well plates with 50% confluency and transfected 24 h later with 50 nM of miRNA precursors and BRCA2 MREs according to manufacturer’s instructions. Cells were also transfected with appropriate negative and positive controls in parallel. Oligonucleotide sequences (100 nts) corresponding to the WT predicted target sites/MREs, WT BRCA2 MRE-4, mutated predicted binding site (MUT BRCA2 MRE-4) or fully complementary to miR-19a or miR-19b (As-miR-19a, As-miR-19b) were synthesized and augmented to include XhoI and NotI restriction sites (Invitrogen/Thermo Fisher Scientific). Scrambled Vector (100 nts) was designed and used as negative control in parallel (**Supplementary Table S2**). Oligonucleotides were annealed, purified, double-digested with the XhoI and NotI restriction enzymes (New England Biolabs, Ipswich, MA, USA) and cloned into the 3′UTR of Renilla luciferase within the psiCHECK-2 Dual-Luciferase Reporter vector (Promega, Madison, WI, USA) using standard techniques. 48 h after cell transfection with the appropriate psiCHECK-2 vector carrying the cloned sequence of interest, miRNA precursors and controls Renilla and Firefly luciferase levels were measured with the Dual-Luciferase Reporter Assay System (Promega) according to the manufacturer’s instructions. Luciferase activity for each sample was measured using a Multi-mode microplate reader Synergy 2 (BioTeck Instruments, Winooski, VT, USA). For the presented analyses, target Renilla luciferase activity was normalized to control Firefly luciferase activity. All data for luciferase assays are expressed as mean ± SE with a sample size of *n* = 3 for each group. Statistical analyses between test and control group were performed with unpaired two-tailed Student’s *t*-test assuming equal variances. *P*-values ≤ 0.05 were considered statistically significant. ^∗^*P* ≤ 0.05, ^∗∗^*P* ≤ 0.01, ^∗∗∗^*P* ≤ 0.001.

## Results

### MiRNAs from the miR-17/92 Cluster are Predicted to Target *BRCA2* mRNA

**FIGURE 1:**
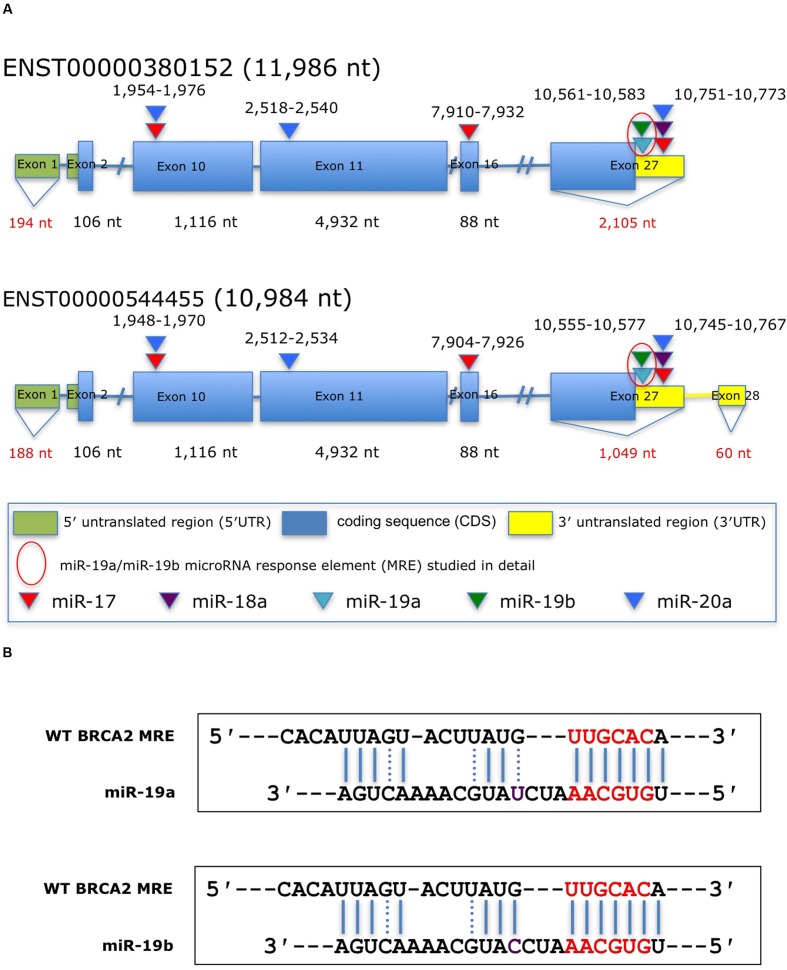
**Predicted miRNAs response elements (MREs) within *BRCA2* for the miR-17/92 cluster.** **(A)** Schematic representation of *BRCA2* transcripts showing the putative miR-17/92 cluster’s MREs in the CDS and the 3′UTR predicted by the RNA22 algorithm and cross-filtered using Ago CLIP-seq-derived heteroduplex architectures. The highlighted MRE for miR-19a/miR-19b was studied in detail. **(B)** The predicted miRNA:mRNA heteroduplexes. Underlined sequences in red are where binding between the miRNA ‘seed’ and the MRE occurs. Purple letter (C or U) is a single different nucleotide between miR-19a and miR-19b. Blue solid lines are Watson–Crick base pairings and dotted blue lines are non-Watson–Crick base pairings, e.g., G:U wobbles.

**FIGURE 2:**
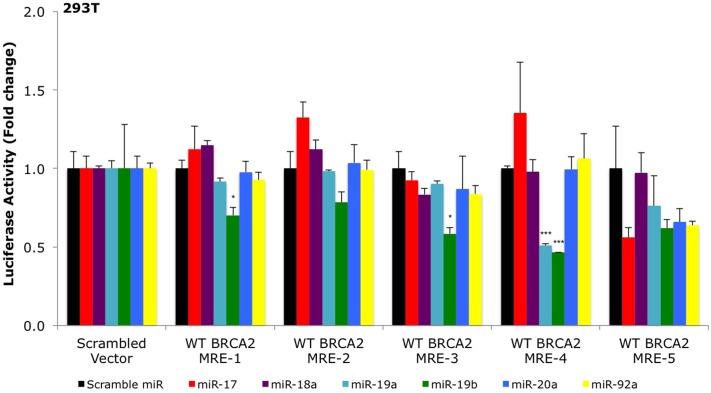
**Primary screening of five putative miR-17/92 MREs by luciferase assays.** The putative wild type *BRCA2* miRNA response elements (WT BRCA2 MREs) were cloned into a luciferase reporter vector psiCHECK-2. Renilla luciferase activity was normalized to Firefly luciferase activity. All shown data are mean ± Standard error. *P*-values ≤ 0.05 were considered statistically significant. ^∗^*P* ≤ 0.05, ^∗∗∗^*P* ≤ 0.001 compared to Scramble miR/Scrambled Vector by two-tailed Student’s *t*-test assuming equal variances, *n* = 3.

To date, only a few miRNAs have been shown to regulate *BRCA2* (Song et al., 2012; Choi et al., 2014), and none of them are members of the miR-17/92 cluster. Using a combination of computational (RNA22) and experimental approaches (Ago CLIP-seq) we selected BRCA2 as one of the targets of the miR-17/92 cluster and identified candidate binding sites within *BRCA2* mRNA (**Figure 1**; **Supplementary Table S3**). Our analysis allowed for mRNA sites that are beyond the 3′UTR and could contain bulges and non-Watson–Crick base pairs in the seed region of the heteroduplex. We identified five sites that were located within the CDS and 3′UTR and screened them using luciferase assays (**Figure 2**). Out of the five putative miRNA binding sites/MREs targeted by four members of the miR-17/92 cluster, WT BRCA2 MRE-4 responded to transfections with miR-19a and miR-19b exhibiting a concomitant statistically significant decrease of luciferase activity (*P* < 0.001). This is the MRE on which we focused our studies. Interestingly, miR-19a and miR-19b are ubiquitously expressed in all tissues and have previously been reported to have oncogenic properties (Mu et al., 2009; Olive et al., 2009; van Haaften and Agami, 2010); this in turn suggests a possible role for these two miRNAs in post-transcriptionally regulating *BRCA2* mRNA.

### MiR-19a and miR-19b Decrease *BRCA2* mRNA Levels

**FIGURE 3:**
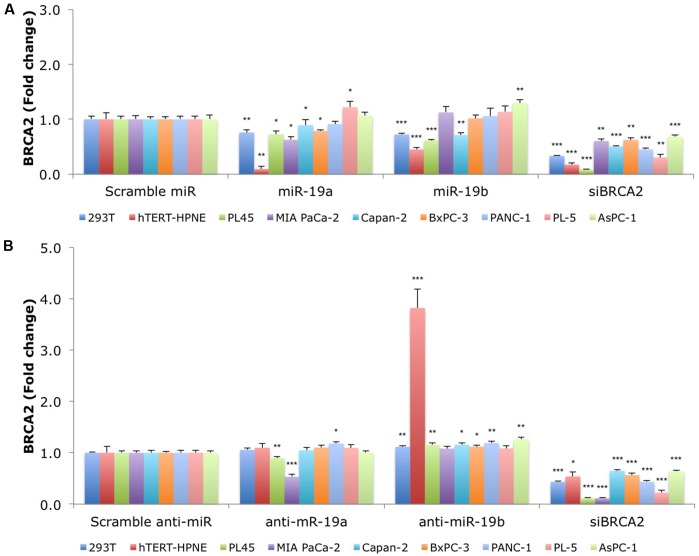
**MiR-19a and miR-19b over-expression affects the endogenous levels of *BRCA2* mRNA in kidney and pancreatic cell lines.** **(A)** Ectopic over-expression of miR-19a/miR-19b decreases *BRCA2* mRNA levels in a first group of nine cell lines. **(B)** Ectopic over-expression of anti-miR-19a/anti-miR-19b increases *BRCA2* mRNA levels in several of the nine cell lines. Cells were transfected with miRNA/anti-miRNA precursors and at 48 h post-transfection the levels of *BRCA2* mRNA were measured using qRT-PCR. Glyceraldehyde-3-phosphate dehydrogenase (*GAPDH*) was used as internal control. All shown data are mean ± Standard error. *P*-values ≤ 0.05 were considered statistically significant. ^∗^*P* ≤ 0.05, ^∗∗^*P* ≤ 0.01, ^∗∗∗^*P* ≤ 0.001 compared to Scramble miR/Scramble anti-miR by two-tailed Student’s *t*-test assuming equal variances, *n* = 3.

We hypothesized that over-expression of miR-19a/miR-19b will decrease endogenous *BRCA2* mRNA levels. Using a panel of 15 cell lines with WT *BRCA2* we examined *BRCA2* mRNA expression following over-expression of each of these two miRNAs. Generally, kidney and pancreatic cell lines showed greater *BRCA2* mRNA suppression compared to breast and colon cell lines. In particular, 293T, hTERT-HPNE, PL45, and Capan-2 cells had the greatest response to both miR-19a and miR-19b treatments ranging from 20 to 80% decrease of *BRCA2* mRNA levels (*P* < 0.05; **Figure 3A**). In contrast, *BRCA2* mRNA level did not change in PANC-1 cells. In MIA PaCa-2 and BxPC-3, *BRCA2* mRNA levels varied only in response to miR-19a treatment, but not to miR-19b. Interestingly, *BRCA2* mRNA levels did not change in response to miR-19a or miR-19b in the breast cancer cell lines MDA-MB-231, MCF7, and BT-20 (**Supplementary Figure S1A**). In addition, *BRCA2* mRNA levels increased in MCF-10A following miR-19a treatment by as much as 2.1x. Also *BRCA2* mRNA levels increased in MDA-MB-468 and HCT 116 following miR-19b treatment. Anti-miR-19a and anti-miR-19b generally abrogated the effects of miR-19a and miR-19b on *BRCA2* mRNA except for MIA PaCa-2 and PL45 cells treated with anti-miR-19a, and MDA-MB-468 and HCT 116 cells treated with anti-miR-19b (**Figure 3B**; **Supplementary Figure S1B**). Furthermore, a siBRCA2 decreased *BRCA2* mRNAs levels in all cell lines (*P* < 0.01) except MDA-MB-468. The summary of the qRT-PCR results in all 15 cell lines are presented in **Supplementary Table S4**. Taken together these results suggest a possible regulation of *BRCA2* mRNA by miR-19a and miR-19b that is dependent upon the cell type, with the greatest effect in pancreatic cancer cells.

### Over-expression of miR-19a and miR-19b Leads to Decrease of BRCA2 Protein Levels

**FIGURE 4:**
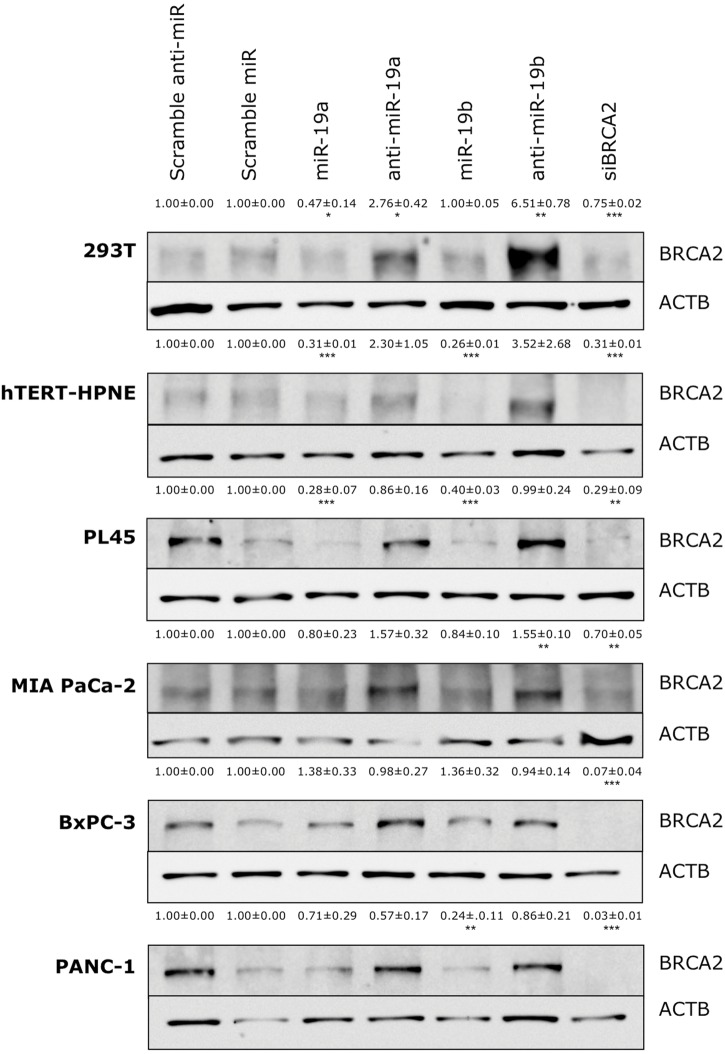
**Ectopic over-expression of miR-19a and miR-19b affects the endogenous levels of BRCA2 protein in kidney and pancreatic cell lines.** Ectopic over-expression of miR-19a/miR-19b decreases BRCA2 protein levels in a cell-dependent manner. Cells were transfected with miRNA/anti-miRNA precursors and at 72 h post-transfection BRCA2 protein was detected by western blot (WB). Band intensities were quantitated using ImageJ. Actin-beta (ACTB) was used as internal control. All shown data are mean ± Standard error. *P*-values ≤ 0.05 were considered statistically significant. ^∗^*P* ≤ 0.05, ^∗∗^*P* ≤ 0.01, ^∗∗∗^*P* ≤ 0.001 compared to Scramble miR/Scramble anti-miR by two-tailed Student’s *t*-test assuming equal variances, *n* = 3.

The above results revealed a specific regulation for *BRCA2* mRNA by miR-19a and miR-19b in six cell lines (293T, hTERT-HPNE, PL45, Capan-2, MIA PACa-2, and BxPC-3), out of the 15 cell lines that we examined. To determine if the decrease in mRNA abundance led to similar decreases in BRCA2 protein levels, we performed BRCA2 WB in those cell lines, under the same conditions, and observed an appreciable decrease of BRCA2 protein levels following treatment with miR-19a and miR-19b in two of the cell lines: hTERT-HPNE and PL45 with band intensity decrease by as much as 74% (*P* < 0.001; **Figure 4**). In 293T and PANC-1 cells BRCA2 protein levels decreased only in response to miR-19a or miR-19b treatment between 53% (*P* < 0.05) and 76% (*P* < 0.01). BRCA2 protein levels were not affected by miR-19a or miR-19b in the BxPC-3 and MIA PaCa-2 pancreatic cancer cell lines. Capan-2 cells showed very low basal level of BRCA2 (result not shown), which could affect result interpretation and were excluded from the subsequent experiments. Over-expression of anti-miR-19a/anti-miR-19b precursors rescues BRCA2 protein levels in 293T, hTERT-HPNE and PL45 by as much as 6.51-fold (*P* < 0.01). Over-expression of anti-miR-19b precursors rescues BRCA2 protein levels in PANC-1 as well BRCA2 protein level is significantly increased with treatment of anti-miR-19b in MIA PACa-2 cell line by as much as 1.5-fold (*P* < 0.01). As anticipated, siBRCA2 consistently decreased BRCA2 protein levels between 25 and 97% in all cell lines (**Figure 4**). We found that the results for qRT-PCR and WB showing BRCA2 levels change with over-expression of miR-19a and miR-19b are mostly concordant (**Supplementary Table S5**). These results suggest that miR-19a and miR-19b inhibit the translation of *BRCA2* mRNA in a subset of the examined cell lines.

### MiR-19a and miR-19b Directly Target an MRE in *BRCA2*’s 3′UTR in Several Cell Lines

**FIGURE 5:**
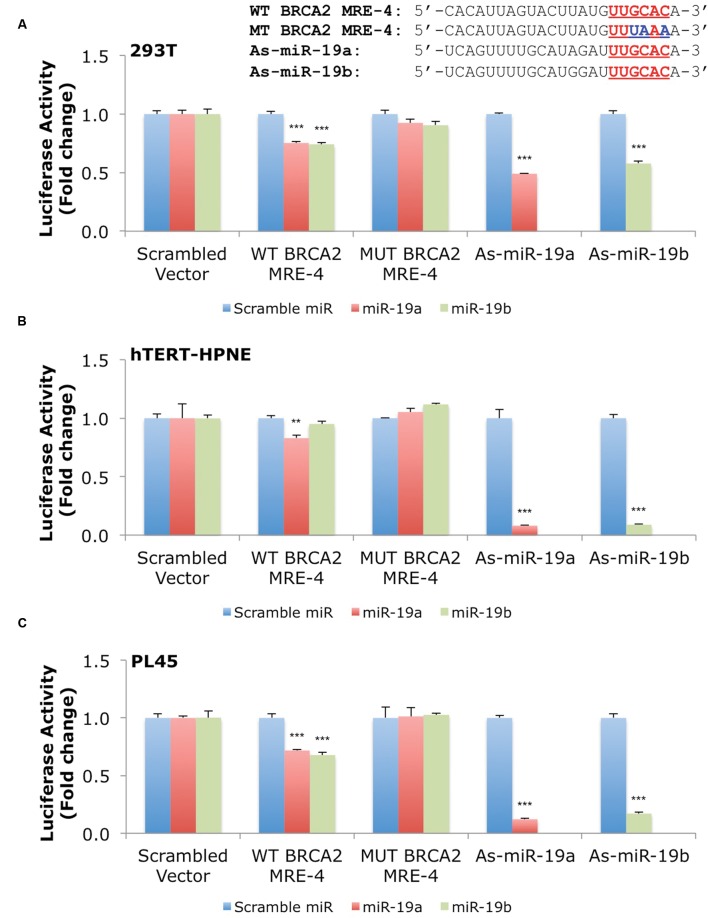
**MiR-19a and miR-19b directly target *BRCA2* within the 3′UTR in several cell lines.** The putative wild type BRCA2 MRE (WT BRCA2 MRE-4) was cloned into a luciferase reporter vector psiCHECK-2 along with mutant BRCA2 MRE (MUT BRCA2 MRE-4) and fully complementary sequences for miR-19a (As-miR-19a) and miR-19b (As-miR-19b). **(A)** 293T, **(B)** hTERT-HPNE, and **(C)** PL45. Underlined sequences in red are where binding between the miRNA ‘seed’ and the MRE occurs. The blue letters are mutated nts in the MRE. Renilla luciferase activity was normalized to Firefly luciferase activity. All shown data are mean ± Standard error. *P*-values ≤ 0.05 were considered statistically significant. ^∗∗^*P* ≤ 0.01, ^∗∗∗^*P* ≤ 0.001 compared to Scramble miR/Scrambled Vector by two-tailed Student’s *t*-test assuming equal variances, *n* = 3.

**FIGURE 6:**
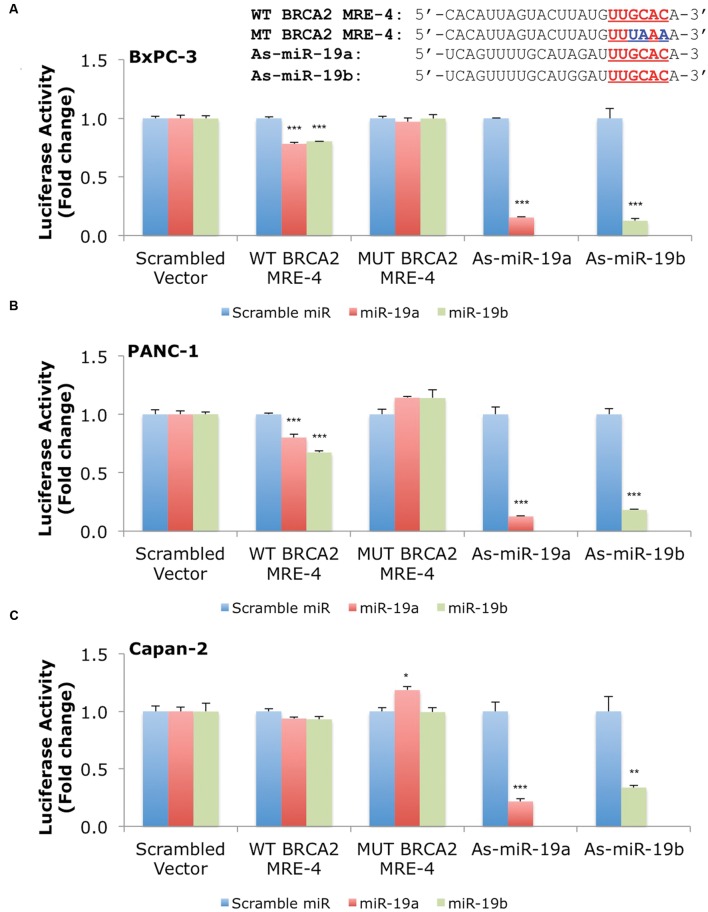
**MiR-19a and miR-19b directly target *BRCA2* within the 3′UTR in additional cell lines.** The putative wild type BRCA2 MRE (WT BRCA2 MRE-4) was cloned into a luciferase reporter vector psiCHEK-2 along with a mutant BRCA2 MRE (MUT BRCA2 MRE-4) and fully complementary sequences for miR-19a (As-miR-19a) and miR-19b (As-miR-19b) **(A)** BxPC-3, **(B)** PANC-1, and **(C)** Capan-2. Underlined sequences in red are where binding between the miRNA ‘seed’ and the MRE occurs. The blue letters are mutated nts in the MRE. Renilla luciferase activity was normalized to Firefly luciferase activity. All shown data are mean ± Standard error. *P*-values ≤ 0.05 were considered statistically significant. ^∗^*P* ≤ 0.05, ^∗∗^*P* ≤ 0.01, ^∗∗∗^*P* ≤ 0.001 compared to Scramble miR/Scrambled Vector by two-tailed Student’s *t*-test assuming equal variances, *n* = 3.

Our results show that miR-19a and miR-19b affect *BRCA2* mRNA and protein abundances in specific cell contexts. To determine whether these effects are due to direct interactions between the miRNAs and *BRCA2*, we performed Dual-Luciferase Reporter Assays. We used constructs that contained the predicted WT target site WT BRCA2 MRE-4, mutant binding site with three consecutive point mutations MUT BRCA2 MRE-4, and ones that are fully complementary to miR-19a (As-miR-19a) and miR-19b (As-miR-19b; **Supplementary Table S2**). These constructs were cloned into the 3′UTR of the luciferase gene of the reporter expression vector and tested in a set of six cell lines: 293T, hTERT-HPNE, PL45, Capan-2, BxPC-3, and PANC-1. Treatments with miR-19a/miR-19b precursors and WT BRCA2 MRE-4 demonstrated significant decrease in luciferase activity, by as much as ∼25% (*P* < 0.001) in 293T, PL45, BxPC-3, and PANC-1 cell lines (**Figures 5** and **6**). Interestingly, in hTERT-HPNE we observed a decrease in luciferase activity only with miR-19a and WT BRCA2 MRE-4 treatments, while in Capan-2 we did not observe a statistically significant reduction of luciferase activity with miR-19a/miR-19b treatments (**Figure 6**). MiR-19a/miR-19b co-transfected with a reporter expression vector containing As-miR-19a/As-miR-19b decreased luciferase levels even further compared to control co-transfection: decreases as much as 50% (*P* < 0.001) in 293T and as much as 80–90% (*P* < 0.001) in other cell lines (**Figure 5**). Point mutations in the miR-19a/miR-19b binding site abolished the inhibitory effect on luciferase activity. This effect was observed for a mutant construct with three consecutive point mutations compared to the WT site. The greatest effect of increased luciferase activity compared to scramble control miRNA precursor was observed in Capan-2 cells: treatment with miR-19a co-transfected with MUT BRCA2 MRE-4 lead to a ∼20% increase (*P* < 0.05) of luciferase activity (**Figure 6**). These observations show that miR-19a and miR-19b directly interact with *BRCA2*, and that this effect is happening in a cell type specific manner similarly to what we showed previously for BRCA1 (Quann et al., 2015).

## Discussion

*BRCA2* is an important gene in the DNA repair pathway whose altered function has been associated to many different cancers (Couch et al., 2007; Cancer Genome Atlas Research Network, 2011, Cancer Genome Atlas Network, 2012; Roy et al., 2012; Beltran et al., 2013; Holter et al., 2015; Norris et al., 2015; Patch et al., 2015). Also, it is known that BRCA2 dysregulation can result in a BRCAness phenotype (Lord and Ashworth, 2016). While the roles of germline and somatic mutations in the gene’s activities have been well characterized, very little is known about its post-transcriptional regulation program (Song et al., 2012; Choi et al., 2014). MiR-19a and miR-19b are ubiquitously expressed and have been shown to be sufficient in capturing the miR-17/92 cluster’s oncogenic properties (Mu et al., 2009; Olive et al., 2009; van Haaften and Agami, 2010). In the above, we examined the interplay between these two competing molecular classes. Specifically, we studied the miRNA-mediated post-transcriptional regulation of *BRCA2* and determined that miR-19a and miR-19b directly interact with an MRE in *BRCA2’s* 3′UTR. Several lines of evidence show that these interactions result in a decrease of *BRCA2* mRNA and protein abundance.

MiR-19a and miR-19b are known to play key roles in the oncogenic properties of the miR-17/92 cluster and are often up-regulated in a wide variety of cancers, including breast and pancreatic cancers (Volinia et al., 2006, 2010; Szafranska et al., 2007; Mogilyansky and Rigoutsos, 2013). Many of the experimentally validated targets (e.g., *PTEN*, *STAT2*, *TSP-1*, and *CTGF*) of these two miRNAs are involved in such processes as cell death, proliferation, immune response and angiogenesis (Ventura et al., 2008; Dews et al., 2010; Mogilyansky and Rigoutsos, 2013). However, to date only a few miRNAs have been shown to target genes in the DNA repair pathway, and in particular *BRCA2* (Hu and Gatti, 2011; Wan et al., 2011; Chowdhury et al., 2013). The ubiquitous expression of *BRCA2* and its long 3′UTR (∼1,500 nts) make the study of its miRNA post-transcriptional regulation a uniquely attractive and challenging task. Over-expression of miR-19a/miR-19b often correlates with loss of BRCA1/BRCA2 protein expression in breast, ovarian cancer, pancreatic, and other cancers (King, 2004; Malone et al., 2009; Li et al., 2010), which raised the possibility that miR-19a/miR-19b could be involved in the regulation of *BRCA2*; however, we are not aware of any earlier reports that demonstrated direct targeting of *BRCA2* mRNA by members of the miR-17/92 cluster and regulation of its mRNA and protein abundance.

Our studies present several lines of evidence that support the direct targeting of *BRCA2* by miR-19a and miR-19b. First, over-expression of each of the two miRNAs results in a decrease of *BRCA2* mRNA and protein levels in a cell-dependent manner. Second, by using a luciferase construct containing the miR-19a/miR-19b MRE we show a decrease in luciferase expression and prove miR-19a/miR-19b and BRCA2 MRE direct physical interactions.

We tested miR-19a/miR-19b:*BRCA2* interactions in 15 cell lines derived from pancreatic, breast, colon cancers, and kidney. All tested cell lines are WT for *BRCA2*, but have mutations in other genes that are implicated in BRCAness. For example, AsPC-1, BxPC-3, MIA PaCa-2, MDA-MB-231, MDA-MB-468, and BT-20 have *TP53* mutations. MDA-MB-468 has a *PTEN* mutation, which has been correlated with HDR defects, but whose exact mechanism is not clear. In addition, a subset of the cell lines has mutations in genes that were previously linked to a cancer phenotype, including *KRAS* (in MDA-MB-231, MIA PaCa-2, Capan-2, AsPC-1, and HCT 116) and *CDKN2A* (in MCF7, MDA-MB-231, and HCT 116).

Recent reports claim that the BRCAness phenotype occurs not only in a large percentage of breast and ovarian cancers, but also in a wide spectrum of cancers, including prostate and pancreatic cancers (Couch et al., 2007; Cancer Genome Atlas Research Network, 2011, Banerji et al., 2012; Cancer Genome Atlas Network, 2012; Stephens et al., 2012; Beltran et al., 2013; Holter et al., 2015; Patch et al., 2015). The BRCAness phenotype could be explained in part by miRNA (dys-) regulation of *BRCA2*. Previous studies linked *BRCA2*’s post-transcriptional regulation by miRNAs to breast and ovarian cancer cell lines (Song et al., 2012; Choi et al., 2014). In this study, we extended the analysis to include kidney, pancreatic, and colon cell lines.

We identified an MRE for miR-19a/miR-19b that is located in the 3′UTR of *BRCA2*’s two protein-coding transcripts. The predicted heteroduplexes with miR-19a or miR-19b comprise classical Watson–Crick base pairing in the seed region of the respective miRNA. However, the identified MRE is not conserved across species e.g., it is present in chimpanzee *(Pan troglodytes)*, but absent in mouse (*Musculus*), chicken *(Gallus gallus domesticus)* and zebrafish *(Danio rerio)*. Of note, our group recently reported the regulation of another DNA repair gene, BRCA1, by the miR-15/107 group of miRNAs, through an MRE located within the mRNA’s CDS; that interaction supported the expanded model of miRNA targeting (Quann et al., 2015).

We studied the impact of ectopic over-expression of miR-19a/miR-19b on the levels of *BRCA2* mRNA and protein in kidney, pancreatic, breast, and colon cells. Studies have shown that triple negative breast cancers (TNBCs) have enriched HDR defects, namely defective RAD51 formation, and increased chromosomal aberrations (Banerji et al., 2012; Cancer Genome Atlas Network, 2012; Stephens et al., 2012). However, our analyses do not support the possibility of *BRCA2* down-regulation by miR-19a or miR-19b in the three TNBC cell lines that we tested, namely MDA-MB-231, MDA-MB-468, and BT-20. Quite paradoxically, we observed the opposite in the MDA-MB-468 cell line: *BRCA2* mRNA levels *increased* following treatment with miR-19b. Possible explanations include increased stabilization of the mRNA leading to increased transcription (Li et al., 2006; Miranda et al., 2006; Huang et al., 2012), the presence of indirect effects on *BRCA2* through, e.g., competitive endogenous RNAs (ceRNAs) interactions (Karreth et al., 2011; Tay et al., 2011) or, a direct interaction with *BRCA2’s* promoter region (Place et al., 2008). Another factor could be the difference in basal levels of *BRCA2* mRNA expression. In fact, MDA-MB-468 cells had the highest level of *BRCA2* expression among breast cancer cells and did not respond to siBRCA2, even though the other cell lines did respond.

Pancreatic cells (hTERT-HPNE, PL45, Capan-2, and BxPC-3) showed, generally, a decrease of *BRCA2* levels upon treatment with miRNAs. Nonetheless, we observed considerable differences among pancreatic cell lines with regard to changes of *BRCA2* mRNA and protein levels following miR-19a/miR-19b treatments. In particular, in BxPC-3 cells, miR-19a treatment led to a decrease of *BRCA2* mRNA but no concomitant change of BRCA2 protein level whereas miR-19b treatment did not change mRNA levels yet led to a large increase of protein abundance. Several previous studies described similar behavior between mRNA and protein levels in the BRCA2 context (Song et al., 2012; Choi et al., 2014) and elsewhere (Schwanhausser et al., 2011; Vogel and Marcotte, 2012; Londin et al., 2014). The two known *BRCA2* mRNAs are rather long (Venkitaraman, 2002; Roy et al., 2012): thus, it is conceivable that cooperative action by more than one miRNAs operating through multiple MREs is needed in order to appreciably decrease *BRCA2* mRNA levels.

We notice that most of the studied pancreatic cell lines (PL45, MIA PaCa-2, Capan-2, BxPC-3, PANC-1, and PL-5) derived from the primary tumor with exception of AsPC-1. However, most of the studied breast cell lines (MDA-MB-231, MDA-MB-468, and MCF7) derived from metastatic sites with the exception of BT-20, which derived from a primary site. Indeed, we observed that miR-19a/miR-19b were able to decrease *BRCA2* mRNA levels from the group of pancreatic cells derived from a primary site, but we didn’t see the same effect in breast cells derived from a primary site.

We presented evidence of direct interactions between miR-19a/miR-19b and the BRCA2 MREs, which were cloned in a luciferase reporter vector. The luciferase assay shows consistent and robust decrease of luciferase activity following treatment with miR-19a and miR-19b in several cell lines (293T, hTERT-HPNE, PL45, BxPC-3, and PANC-1).

The main finding of this work is the discovery of a link between miR-19a and miR-19b, two members of the oncogenic miR-17/92 cluster, and the post-transcriptional regulation of *BRCA2*, one of the regulators of the DDR pathway. The functional implications of this link are not known and need to be investigated. In particular, it will be important to determine whether these interactions lead to defective HDR, a hallmark of BRCAness. The results would improve our understanding of BRCA2’s post-transcriptional regulation by miRNAs and could prove helpful in improving clinical management of the disease and designing improved therapies that target the DNA repair defects. For example, in the presence of BRCAness, a tumor with a miRNA down-regulated BRCA2 can have similar clinical features as a tumor with *BRCA2* germline mutations: in such a case, patients from either group would have similar prognosis and may require similar adjuvant or neoadjuvant therapies. Also tumors with miRNA-down-regulated *BRCA2* could be more sensitive to platinum salts and PARP inhibitors, which would help expand the treatment options for patients with sporadic cancers.

Members of the miR-17/92 cluster have been shown to target a number of important genes, but its involvement in DNA repair awaits further investigation. Given the wide array of cancers in which miR-19a and miR-19b are dys-regulated such studies are warranted. Lastly, it is important to remain aware of the cell-type dependence that our experiments uncovered and which mirror findings we reported recently for BRCA1 (Quann et al., 2015). This dependence suggests a very rich molecular context in which regulation of BRCA2 takes place and whose details await discovery and characterization.

## Author Contributions

EM, YJ, and IR designed the experiments. EM and YJ performed the experiments. KQ and HZ helped with technical advices and selected constructs preparation. PC performed computational work. EM, EL, and IR analyzed the data. EM and IR wrote the manuscript. IR conceived and supervised the project. All authors have reviewed and approved the final manuscript.

## Conflict of Interest Statement

The authors declare that the research was conducted in the absence of any commercial or financial relationships that could be construed as a potential conflict of interest.

## Abbreviations

3′UTR 3′: untranslated region
5′UTR 5′: untranslated region
ACTB: actin-beta
Ago: argonaute protein
Ago CLIP-seq: argonaute cross-linking immunoprecipitation and deep sequencing
ATCC: American type culture collection
BRCA1: breast cancer 1, early onset gene
BRCA2: breast cancer 2, early onset gene
*C. elegans*: *Caenorhabditis elegans;*
cDNA: complementary DNA
CDS: coding sequence
ceRNAs: competitive endogenous RNAs
DNA: deoxyribonucleic acid
DDR: DNA damage response
DSDB: double strand DNA breaks
GAPDH: glyceraldehyde 3-phosphate dehydrogenase
HDR: homology-directed repair
miRNA: microRNA
mRNA: messenger ribonucleic acid
MRE: miRNA response element
MUT: mutant type
ncRNA: non-coding RNA
nts: nucleotides
PARP: poly (ADP ribose) polymerase
qRT-PCR: quantitative reverse-transcription polymerase chain reaction
RNA: ribonucleic acid
RNA-seq: RNA sequencing
SE: standard error
SDS-PAGE: sodium dodecyl sulfate polyacrylamide gel electrophoresis
TNBC: triple negative breast cancer
UTR: untranslated region
WB: western blot
WT: wild type
