# Supplementary material for: Post-transcriptional Regulation of BRCA2 through Interactions with miR-19a and miR-19b
Source: Front Genet. 2016 Aug 31;7:143. doi: 10.3389/fgene.2016.00143 (PMC5005319; doi:10.3389/fgene.2016.00143)
Supplement: Supplementary file 1 [file Presentation_5.PDF]

SUPPLEMENTARY MATERIAL

Post-transcriptional Regulation of BRCA2 through Interactions with miR-19a and miR-19b

Elena Mogilyansky[1], Peter Clark[2], Kevin Quann[3], Honglei Zhou[1], Eric Londin[1], Yi Jing[1], and Isidore Rigoutsos[1,*]

[1]Computational Medicine Center, Sidney Kimmel Medical College, Thomas Jefferson University, 1020 Locust Street, Suite M-81, Philadelphia, PA 19107, USA
[2]Department of Pathology and Laboratory Medicine, The Children's Hospital of Philadelphia, 3401 Civic Center Boulevard, Philadelphia, PA 19104, USA
[3]Sidney Kimmel Medical College, Thomas Jefferson University, 1025 Walnut Street, Suite #100, Philadelphia, PA 19107, USA

* To whom correspondence should be addressed. Tel: +1-(215) 503-6152; Fax: +1-(215) 503-0466; Email: isidore.rigoutsos@jefferson.edu

Abbreviations

3´UTR	3´ untranslated region
Ago	argonaute protein
Ago CLIP-Seq	argonaute cross-linking immunoprecipitation and deep sequencing
As	antisense
BRCA2	breast cancer 2, early onset gene
CDS	coding sequence
FWD	forward
GAPDH	glyceraldehyde 3-phosphate dehydrogenase
miRNA	microRNA
MRE	miRNA response element
MUT	mutant type
nts	nucleotides
qRT-PCR	quantitative reverse-transcription polymerase chain reaction
RVS	reverse
SE	standard error
UTR	untranslated region
WB	western blot
WT	wild type

Supplementary Figures and Tables Legends

Supplementary Figure S1. MiR-19a and miR-19b over-expression affects the endogenous levels of *BRCA2* mRNA in breast and colon cell lines. (A) Ectopic over-expression of miR-19a/miR-19b increases *BRCA2* mRNA levels in a second group of six cell lines**. (B)** Ectopic over-expression of anti-miR-19a/anti-miR-19b abrogates the effect of miR-19a/miR-19b on *BRCA2* mRNA levels in several of the six cell lines. Cells were transfected with miRNA/anti-miRNA precursors and at 48 h post-transfection the levels of *BRCA2* mRNA were measured using qRT-PCR. Glyceraldehyde-3-phosphate dehydrogenase *(GAPDH)* was used as internal control. All shown data are mean ± Standard error. *P*-values ≤ 0.05 were considered statistically significant. **P* ≤ 0.05, ***P* ≤ 0.01, ****P* ≤ 0.001 compared to Scramble miR/Scramble anti-miR by two-tailed Student's t-test assuming equal variances, *n = 3*.

Supplementary Table S1. Sequences of the primers used for qRT-PCR. Primers for *BRCA2* were adapted from Choi et al., *eLife*, 2014.

Supplementary Table S2. Sequences of the oligonucleotides for BRCA2 MREs targeted by miR-19a/miR-19b. BRCA2 MREs were synthesized as oligonucleotides (100 nts; forward: include actual MRE and reverse-complementary to MRE) by Invitrogen (Thermo Fisher Scientific). Each oligonucleotide includes the actual MRE (23 nts) (red letters, underlined), adjacent genomic sequences (purple letters) with XhoI (bright green letters) and NotI (orange letters) restriction sites (Invitrogen/Thermo Fisher Scientific) and random nested nts (bright blue letters) at both ends of the oligonucleotide. The binding site/MRE (23 nts) is shown underlined (red letters). WT BRCA2 MRE-1 - WT BRCA2 MRE-5: wild type BRCA2 MREs; MUT BRCA2 MRE-4: mutated BRCA2

MRE (mutated sites in dark blue); As-miR-19a/As-miR-19b: fully complementary sequences to miR-19a and miR-19b (light blue letters) respectively. Oligonucleotides were annealed, purified, double-digested with the XhoI and NotI restriction enzymes (New England BioLabs, Ipswich, MA, USA) and cloned into the 3´UTR of Renilla luciferase within psiCHECK-2 dual luciferase reporter vector (Promega, Madison, WI, USA) using standard techniques.

Supplementary Table S3. Putative binding sites for the miR-17/92 cluster members. The 23-nucleotide sequences were predicted as putative targets for miR-17, miR-18a, miR-19a, miR-19b and miR-20a using the RNA22 algorithm with publicly available and in-house data for Ago CLIP-seq. We identified a total of five binding sites/MREs: three in the CDS (WT BRCA2 MRE-1, WT BRCA2 MRE-2, WT BRCA2 MRE-3) and two in the 3´UTR (WT BRCA2 MRE-4 and WT BRCA2 MRE-5) of BRCA2.

Supplementary Table S4. Summary of *BRCA2* mRNA (qRT-PCR) levels with over-expression of miR-19a/miR-19b and anti-miR-19a/anti-miR-19b in pancreatic, breast, kidney, and colon cell lines. Cells were transfected with miRNA/anti-miRNA precursors and at 48 h post-transfection the levels of *BRCA2* mRNA were measured using qRT-PCR. All shown data are mean ± Standard error. *P*-values ≤ 0.05 were considered statistically significant compared to Scramble miR/Scramble anti-miR by two-tailed Student's t-test assuming equal variances, $n = 3$. Green - statistically significant decrease of *BRCA2* level, red - statistically significant increase of *BRCA2* level, grey - no statistically significant change of *BRCA2* level compared to Scramble miR/Scramble anti-miR.

Supplementary Table S5. Summary of *BRCA2* mRNA (qRT-PCR) and protein (WB) levels with over-expression of miR-19a/miR-19b and anti-miR-19a/anti-miR-19b in pancreatic and kidney cell lines. qRT-PCR: Cells were transfected with miR-19a/miR-19b and anti-miR-19a/anti-miR-19b precursors and at 48 h post-transfection the levels of *BRCA2* mRNA were measured using qRT-PCR. WB: Cells were transfected with miR-19a/miR-19b and anti-miR-19a/anti-miR-19b precursors constructs and at 72 h post-transfection the levels of BRCA2 were measured using WB. Band intensities were measured using ImageJ. qRT-PCR and WB: All shown data are mean ± Standard error. *P*-values ≤ 0.05 were considered statistically significant compared to Scramble miR/Scramble anti-miR by two-tailed Student's t-test assuming equal variances, *n = 3*. Green - statistically significant decrease of BRCA2 level, red - statistically significant increase of BRCA2 level, grey - no statistically significant change of BRCA2 level compared to Scramble miR/Scramble anti-miR.

Supplementary Figures and Tables

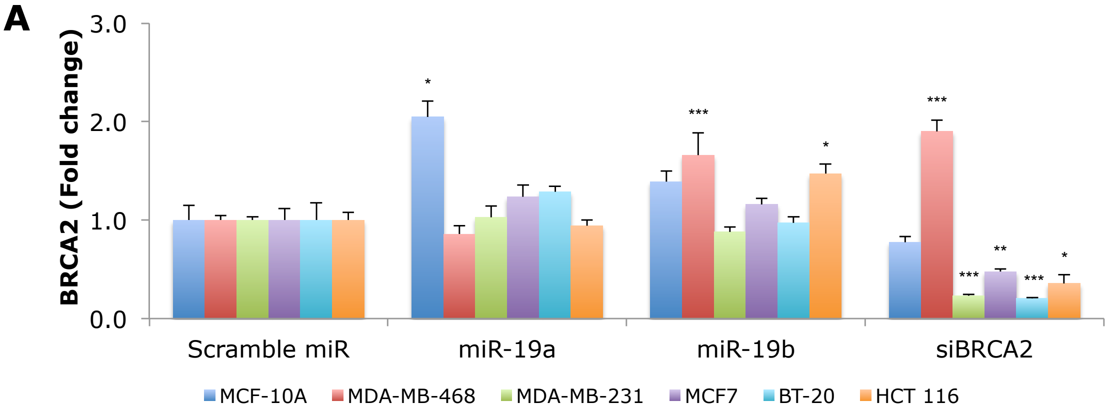

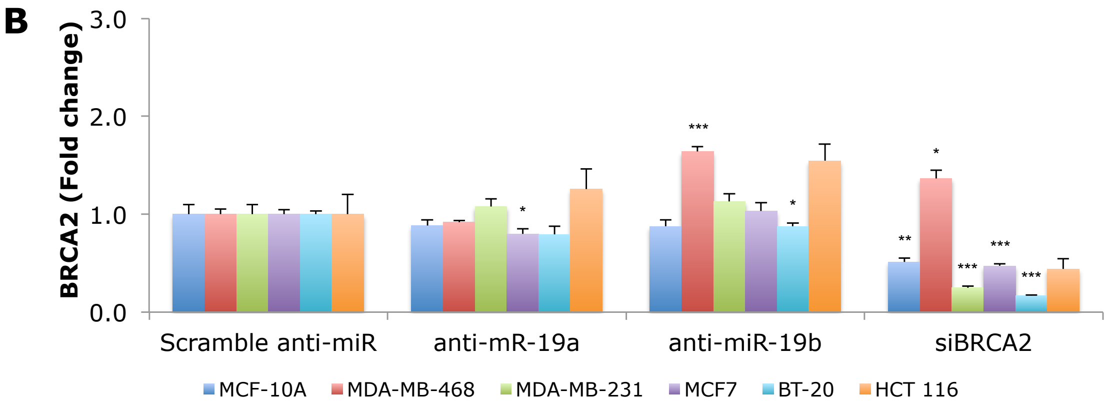

Supplementary Figure S1

Supplementary Table S1

ID	Primer Sequence (5’ - 3’)
BRCA2 – FWD	5′-GCCAAGTCATGCCACACATT-3′
BRCA2 – REV	5′-TGTGCCATCTGGAGTGCTTT-3′
GAPDH – FWD	5′-ATCACCATCTTCCAGGAGCGA-3′
GAPDH – REV	5′-GATGACCCTTTTGGCTCCCC-3′

Supplementary Table S2

MRE ID	Oligonucleotide Sequence (5′ - 3′)
WT BRCA2 MRE-1 – FWD	ATCATTACTCGAGCACAGAATTCTGTAGCTTTGAAGAATGCAGGTTTAATATCCACTTTGAAAAAGAAAACAAATAAGTTTATTTGCGGCCGCATTGTCA
WT BRCA2 MRE-1 – REV	TGACAATGCGGCCGCAAATAAACTTATTTGTTTTCTTTTTCAAAGTGGATATTAAACCTGCATTCTTCAAAGCTACAGAATTCTGTGCTCGAGTAATGAT
WT BRCA2 MRE-2 – FWD	ATCATTACTCGAGAGAAAAGTCTTTTATATGATCATGAAAATGCCAGCACTCTTATTTTAACTCCTACTTCCAAGGATGTTCTGTGCGGCCGCATTGTCA
WT BRCA2 MRE-2 – REV	TGACAATGCGGCCGCACAGAACATCCTTGGAAGTAGGAGTTAAAATAAGAGTGCTGGCATTTTCATGATCATATAAAAGACTTTTCTCTCGAGTAATGAT
WT BRCA2 MRE-3 – FWD	ATCATTACTCGAGCAGCAAAAATGCAGAGTCTTTTCAGTTTCACACTGAAGATTATTTTGGTAAGGAAAGTTTATGGACTGGAAAGCGGCCGCATTGTCA
WT BRCA2 MRE-3 – REV	TGACAATGCGGCCGCTTTCCAGTCCATAAACTTTCCTTACCAAAATAATCTTCAGTGTGAAACTGAAAAGACTCTGCATTTTTGCTGCTCGAGTAATGAT
WT BRCA2 MRE-4 – FWD	ATCATTACTCGAGAGACTGGAATATAATTTCAAACCACACATTAGTACTTATGTTGCACAATGAGAAAAGAAATTAGTTTCAAATGCGGCCGCATTGTCA
WT BRCA2 MRE-4 – REV	TGACAATGCGGCCGCATTTGAAACTAATTTCTTTTCTCATTGTGCAACATAAGTACTAATGTGTGGTTTGAAATTATATTCCAGTCTCTCGAGTAATGAT
WT BRCA2 MRE-5 – FWD	ATCATTACTCGAGATCTTTGGCTGAGCTCGGTGGCTCATGCCTGTAATCCCAACACTTTGAGAAGCTGAGGTGGGAGGAGTGCTTGCGGCCGCATTGTCA
WT BRCA2 MRE-5 – REV	TGACAATGCGGCCGCAAGCACTCCTCCCACCTCAGCTTCTCAAAGTGTTGGGATTACAGGCATGAGCCACCGAGCTCAGCCAAAGATCTCGAGTAATGAT
MUT BRCA2 MRE-4 – FWD	ATCATTACTCGAGAGACTGGAATATAATTTCAAACCACACATTAGTACTTATGTTTAAAAATGAGAAAAGAAATTAGTTTCAAATGCGGCCGCATTGTCA
MUT BRCA2 MRE-4 – REV	TGACAATGCGGCCGCATTTGAAACTAATTTCTTTTCTCATTTTTAAACATAAGTACTAATGTGTGGTTTGAAATTATATTCCAGTCTCTCGAGTAATGAT
As-miR-19a MRE – FWD	ATCATTACTCGAGAGACTGGAATATAATTTCAAACCATCAGTTTTGCATAGATTTGCACAATGAGAAAAGAAATTAGTTTCAAATGCGGCCGCATTGTCA
As-miR-19a MRE – REV	TGACAATGCGGCCGCATTTGAAACTAATTTCTTTTCTCATTGTGCAAATCTATGCAAAACTGATGGTTTGAAATTATATTCCAGTCTCTCGAGTAATGAT
As-miR-19b MRE – FWD	ATCATTACTCGAGAGACTGGAATATAATTTCAAACCATCAGTTTTGCATGGATTTGCACAATGAGAAAAGAAATTAGTTTCAAATGCGGCCGCATTGTCA
As-miR-19b MRE – REV	TGACAATGCGGCCGCATTTGAAACTAATTTCTTTTCTCATTGTGCAAATCCATGCAAAACTGATGGTTTGAAATTATATTCCAGTCTCTCGAGTAATGAT
Scrambled Vector – FWD	ATCATTACTCGAGACAGTCTAGGCATATGCCCTTGCGTGCACTTCGCACATGACTGGTGGCCAGCGCCCAGTCTGGTCGCGTTACGCGGCCGCATTGTCA
Scrambled Vector – REV	TGACAATGCGGCCGCGTAACGCGACCAGACTGGGCGCTGGCCACCAGTCATGTGCGAAGTGCACGCAAGGGCATATGCCTAGACTGTCTCGAGTAATGAT

Supplementary Table S3

MRE ID	Location	Sequence (5′ - 3′)	MiRNAs, which Target MRE
WT BRCA2 MRE-1	CDS/Exon 10	5′-ATGCAGGTTTAATATCCACTTTG-3′	miR-17, miR-20a
WT BRCA2 MRE-2	CDS/Exon 11	5′-AAAATGCCAGCACTCTTATTTTA-3′	miR-20a
WT BRCA2 MRE-3	CDS/Exon 16	5′-GTTTCACACTGAAGATTATTTTG-3′	miR-17
WT BRCA2 MRE-4	3′UTR	5′-CACATTAGTACTTATGTTGCACA-3′	miR-19a, miR-19b
WT BRCA2 MRE-5	3′UTR	5′-ATGCCTGTAATCCCAACACTTTG-3′	miR-17, miR-20a, miR-18a

Supplementary Table S4

#	Cell Line	Test	Scramble anti-miR	Scramble miR	miR-19a	anti-miR-19a	miR-19b	anti-miR-19b	siBRCA2
1	**293T**	qRT-PCR	1.00±0.02	1.00±0.06	0.76±0.05	1.06±0.04	0.74±0.02	1.12±0.02	0.33±0.01
2	**hTERT-HPNE**	qRT-PCR	1.00±0.13	1.00±0.12	0.09±0.05	1.10±0.08	0.45±0.03	3.82±0.37	0.18±0.031
3	**PL45**	qRT-PCR	1.00±0.03	1.00±0.06	0.73±0.06	0.90±0.03	0.62±0.01	1.15±0.04	0.09±0.01
4	**MIA PaCa-2**	qRT-PCR	1.00±0.04	1.00±0.07	0.63±0.06	0.53±0.05	1.13±0.11	1.08±0.04	0.61±0.04
5	**Capan-2**	qRT-PCR	1.00±0.05	1.00±0.05	0.89±0.11	1.05±0.06	0.71±0.04	1.16±0.04	0.50±0.02
6	**BxPC-3**	qRT-PCR	1.00±0.02	1.00±0.05	0.79±0.03	1.10±0.04	1.02±0.06	1.12±0.03	0.62±0.04
7	**PANC-1**	qRT-PCR	1.00±0.05	1.00±0.06	0.91±0.05	1.18±0.04	1.06±0.14	1.19±0.03	0.45±0.02
8	**PL-5**	qRT-PCR	1.00±0.05	1.00±0.06	1.22±0.18	1.10±0.06	1.14±0.11	1.09±0.05	0.30±0.06
9	**AspC-1**	qRT-PCR	1.00±0.03	1.00±0.08	1.07±0.06	1.01±0.03	1.29±0.07	1.26±0.04	0.69±0.03
10	**MCF-10A**	qRT-PCR	1.00±0.10	1.00±0.15	2.05±0.16	0.89±0.06	1.39±0.11	0.88±0.07	0.77±0.06
11	**MDA-MB-468**	qRT-PCR	1.00±0.06	1.00±0.05	0.86±0.09	0.92±0.02	1.66±0.23	1.64±0.05	1.90±0.12
12	**MDA-MB-231**	qRT-PCR	1.00±0.10	1.00±0.04	1.03±0.12	1.08±0.08	0.88±0.05	1.13±0.08	0.23±0.02
13	**MCF7**	qRT-PCR	1.00±0.05	1.00±0.12	1.24±0.12	0.80±0.05	1.16±0.06	1.03±0.09	0.48±0.03
14	**BT-20**	qRT-PCR	1.00±0.03	1.00±18	1.29±0.06	0.80±0.08	0.97±0.06	0.88±0.04	0.21±0.01
15	**HCT 116**	qRT-PCR	1.00±0.20	1.00±0.08	0.94±0.06	1.26±0.21	1.47±0.10	1.55±0.17	0.36±0.09

Supplementary Table S5

#	Cell Line	Test	Scramble anti-miR	Scramble miR	miR-19a	anti-miR-19a	miR-19b	anti-miR-19b	siBRCA2
1	**293T**	qRT-PCR	1.00±0.02	1.00±0.06	0.76±0.05	1.06±0.04	0.74±0.02	1.12±0.02	0.33±0.01
		WB	1.00±0.00	1.00±0.00	0.47±0.14	2.76±0.42	1.00±0.05	6.51±0.78	0.75±0.02
2	**hTERT-HPNE**	qRT-PCR	1.00±0.13	1.00±0.12	0.09±0.05	1.10±0.08	0.45±0.03	3.82±0.37	0.18±0.031
		WB	1.00±0.00	1.00±0.00	0.31±0.01	2.30±1.05	0.26±0.01	3.52±2.68	0.31±0.01
3	**PL45**	qRT-PCR	1.00±0.03	1.00±0.06	0.73±0.06	0.90±0.03	0.62±0.01	1.15±0.04	0.09±0.01
		WB	1.00±0.00	1.00±0.00	0.28±0.07	0.86±0.16	0.40±0.03	0.99±0.24	0.29±0.09
4	**MIA PaCa-2**	qRT-PCR	1.00±0.04	1.00±0.07	0.63±0.06	0.53±0.05	1.13±0.11	1.08±0.04	0.61±0.04
		WB	1.00±0.00	1.00±0.00	0.80±0.23	1.57±0.32	0.84±0.10	1.55±0.10	0.70±0.05
5	**BxPC-3**	qRT-PCR	1.00±0.02	1.00±0.05	0.79±0.03	1.10±0.04	1.02±0.06	1.12±0.03	0.62±0.04
		WB	1.00±0.00	1.00±0.00	1.38±0.33	0.98±0.27	1.36±0.32	0.94±0.14	0.07±0.04
6	**PANC-1**	qRT-PCR	1.00±0.05	1.00±0.06	0.91±0.05	1.18±0.04	1.06±0.14	1.19±0.03	0.45±0.02
		WB	1.00±0.00	1.00±0.00	0.71±0.29	0.57±0.17	0.24±0.11	0.86±0.21	0.03±0.01
